# An Evolutionary View of Tiger Conservation

**DOI:** 10.1371/journal.pbio.0020453

**Published:** 2004-12-07

Tyger! Tyger! burning bright

In the forests of the night

What immortal hand or eye

Could frame thy fearful symmetry?

When William Blake wrote these words in the late 1700s, the deforestation and habitat destruction that would decimate wild tiger populations had already begun. In 1900, an estimated 100,000 wild tigers lived throughout much of Asia, from India in the west to Sumatra and Indonesia in the south to Siberia in the east. Today, the ongoing stresses of habitat loss, hunting, and an illegal trade in tiger parts have spared fewer than 7,000 tigers. Of eight traditionally classified subspecies of *Panthera tigris*, three have gone extinct since the 1940s.

Conservation strategies to combat this grinding attrition are tailored to each subspecies. But several lines of evidence suggest that subspecies designations—based on geographic range and morphological traits such as body size, skull traits, coat color, and striping patterns—may be flawed. An earlier molecular study of 28 tigers found little evidence of genetically distinct subspecies, while surveys of tiger habitat found few physical barriers sufficient for subspecies isolation.

To get a clearer picture of the genetic structure of existing tiger populations, Shu-Jin Luo, Jae-Heup Kim, Stephen O'Brien, and nineteen colleagues performed a comprehensive genetic analysis of mitochondrial and nuclear genes from over 130 tigers. By identifying distinct patterns of variation within these gene families, the authors reconstructed the evolutionary distribution and ancestry of the tiger. Their results support many of the traditional subspecies designations and identify further subdivisions in others.[Fig pbio-0020453-g001]

**Figure pbio-0020453-g001:**
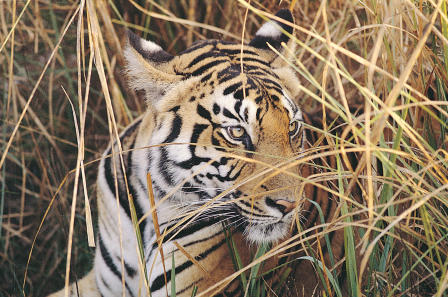
A Bengal tiger in the tall grassland (Photo: Ullas Karanth, WCS-India)

Luo et al. collected “voucher specimens” (taken from animals of verified wild ancestry and geographic origin) of blood, skin, and hair from 134 tigers representing the entire tiger range, and examined them, along with samples of preserved pelts and hair, for three molecular markers. The markers—a stretch of mitochondrial DNA (mtDNA) sequence, a gene with highly variable DNA sequence called *DRB* that's involved in pathogen recognition, and short repeating genetic elements called microsatellites—act as unique signposts that flag significant demographic and evolutionary events in the tiger populations.

mtDNA sequences were extracted from tigers originating in the Russian far east (Siberian, or Amur, tigers), south China, northern Indochina, the Malaya Peninsula, Sumatra, and the Indian subcontinent. The mtDNA analysis identified 30 haplotypes—characteristic regions on a chromosome—that could be clustered. Some of the clusters supported traditional classifications—e.g., for the Sumatran (*P. t. sumatrae*) and (*P. t. tigris*) Bengal tigers—but others suggested that the Indochinese subspecies (*P. t. corbetti*) should be divided into two groups, representing a northern Indochinese and a peninsular Malaya population (which the authors designated respectively as *P. t. corbetti* and *P. t. jacksoni*, after the tiger conservationist Peter Jackson). Interestingly, clusters for the captive South China tigers also fell into two distinct lineages—*P. t. amoyensis*, the traditional grouping, and *P. t. corbetti*, though the designation is still tentative. These subdivisions were largely supported by the other genetic analyses.

The distinct genetic patterns found in the tiger populations suggest six rather than five living subspecies. Reduced gene flow and genetic drift in isolated populations, as well as human activity, likely caused these partitions. The low genetic variability seen in the Siberian tigers, for example, might be explained by severe population declines: the animals were nearly exterminated in the early 1900s, and today only 500 remain. Sumatran tigers, on the other hand, show relatively high genetic variability and uniqueness, possibly reflecting a historically large breeding population that was later isolated.

Whether recent population and habitat declines, as opposed to earlier events, can fully explain these patterns is not clear. But these results offer valuable data for conservation strategies and captive breeding programs that rely on distinctions in subspecies taxonomy and geographic provenance. Evoking both the darker side of creation and humanity, Blake could not have imagined the modern fate of his “Tyger.” Scholars have long debated the multilayered meaning of his poem, including the second stanza, which starts,

In what distant deeps or skies

Burnt the fire of thine eyes?

Will we reduce future generations to a literal reading?

